# Incidence and survival outcomes of myocarditis and pericardial diseases associated with immune checkpoint inhibitor therapy

**DOI:** 10.1186/s40959-025-00300-1

**Published:** 2025-03-05

**Authors:** Aya F. Ozaki, Michael Sayer, Hirofumi Hamano, Misako Nagasaka, Benjamin J. Lee, Jean Doh, Ali Naqvi, Nareh Nowrouzi, Yoshito Zamami, Pranav M. Patel

**Affiliations:** 1 School of Pharmacy & Pharmaceutical Sciences, University of California, 802 W Peltason Dr, Room 106A, Irvine, CA 92617 USA; 2 Department of Pharmacy, Okayama University Hospital, Okayama, Japan; 3 Division of Hematology and Oncology, University of California, Irvine, CA USA; 4 Department of Pharmacy, University of California, Irvine, CA USA; 5 Division of Cardiology, Department of Medicine, University of California, Irvine, CA USA

**Keywords:** Immune checkpoint inhibitors, Myocarditis, Cardiotoxicity, Immune-related adverse events, Overall survival

## Abstract

**Introduction:**

Immune checkpoint inhibitor(ICI) induced cardiac immune related adverse events are challenging to study; Leveraging large data bases like TriNetX global health network may provide needed insights.

**Methods:**

We performed a retrospective cohort study including patients diagnosed neoplasm and 18 and older when receiving ICI therapy from 1/1/2011 to 12/31/2022. Queried ICD 9/10 codes identified patients experiencing myocarditis, pericarditis, pericardial effusion, and cardiac tamponade within 1 year of ICI initiation. Survival analyses compared one-year overall survival (OS) of patients experiencing cardiac irAEs against propensity score matched populations not experiencing them.

**Results:**

In 88,928 identified ICI patients, the incidence of myocarditis(0.48%), pericarditis(0.22%), and cardiac tamponade(0.47%) were less than 1% while pericardial effusion occurred in 4.71% of patients. Hazard ratios (HRs) were significantly higher in all cardiac irAE groups: myocarditis (HR:1.26, 95% CI:1.04–1.54, *p* = 0.02), pericarditis (HR:1.36, 95% CI:1.02–1.82, *p* = 0.04), pericardial effusion (HR:1.49, 95% CI:1.39–1.59, *p* < 0.0001), cardiac tamponade (HR:2.15, 95% CI:1.79–2.57, *p* < 0.0001), and overall pericardial disease (HR:1.46, 95% CI:1.37–1.56, *p* < 0.0001). There was no significant difference in OS between myocarditis and pericarditis or overall pericardial diseases.

**Discussion/conclusion:**

Utilizing a uniquely large cohort of ICI patients, this study further shows the rarity of cardiac inflammatory irAEs and highlights their significant impact on patient survival.

**Supplementary Information:**

The online version contains supplementary material available at 10.1186/s40959-025-00300-1.

## Highlights

• Leveraging TriNetX global health network, we identified a cohort of 88,928 patients receiving immune checkpoint inhibitors

• Cardiac inflammatory events including myocarditis, pericarditis, and cardiac tamponade occurred in less than l% of patients

• Pericardial effusion was more common, occurring in 4.71% of patients

• All cardiac inflammatory events significantly reduced 1-year overall survival compared to matched controls

• Patients having pericardium-based events had similar survival outcomes to myocarditis

**Supplementary Information:**

The online version contains supplementary material available at 10.1186/s40959-025-00300-1.

## Introduction

Immune checkpoint inhibitors (ICIs) have revolutionized cancer treatment by significantly improving mortality rates across various malignancies. However, ICIs can induce a wide range of immune-related adverse events (irAEs) [1]. Among these, cardiac inflammatory adverse events are particularly severe but less understood due to their infrequent presentation [2]. Previous studies have been limited by small sample sizes, largely due to low incidence of conditions such as myocarditis [3]. Furthermore, the spectrum of ICI-induced pericarditis and other pericardial diseases remains understudied. These cardiac inflammatory adverse events (AEs) present with various clinical manifestations, contributing to uncertainty surrounding their clinical impact on cancer patients [4]. Addressing this gap, our study assessed the incidence and overall survival outcomes associated with myocarditis and pericardial diseases in patients undergoing ICI therapy, offering insights into the management and prognosis of these potentially life-threatening complications.

## Methods

We conducted a retrospective cohort analysis utilizing the TriNetX platform, a global health research network that aggregates real-world data from various healthcare organizations. Our patient population included those with a diagnosed neoplasm and 18 and older when receiving ICI therapy from 1/1/2011 to 12/31/2022. First, utilizing ICD 9/10 codes we identified patients experiencing myocarditis, pericarditis, pericardial effusion, and cardiac tamponade post-ICI initiation (Table 1). We than determined the incidence of these events within the ICI patient population. Incidence calculations for cardiac irAEs excluded patients having any diagnosis history of each event (based on used ICD 9 and 10 codes to identify cardiac irAEs) prior to ICI initiation, to eliminate confounding from past medical histories with cardiac inflammation prior to ICI initiation.

**Table 1:** ICD 9/10 codes utilized to identify Cardiac inflammatory adverse events. From left to write, the first column represents the of the cardiac inflammatory adverse event type, followed by ICD-9 codes and ICD-10 codes used to identify them within trinetx data base

	**ICD 9 Codes**	**ICD 10 Codes**
Myocarditis	422.0, 422.90, 422.91, 422.93, 422.99, 429.0	I40.1, I40.8, I40.9, I41, I51.4
Pericarditis	420.0, 420.90, 420.91, 420.99	I30.0, I30.8, I30.9, I32
Pericardial Effusion	NA	I31.3, I31.31, I31.39
Cardiac Tamponade	423.3	I31.4
Overall Pericardial Diseases	420.0, 420.91, 420.99, 420.90, 423.3, 423.8, 423.9	I30.0, I30.8, I30.9, I31.2, I31.3, 31.31, I31.39, I31.4, I31.8, I31.9, I32

Following this, we implemented Cox-proportional hazards models and Kaplan–Meier survival curves to compare one-year overall survival (OS) of patients experiencing targeted cardiac inflammatory AEs against those that do not. Then, we compared OS between patients experiencing myocarditis to those experiencing pericarditis or overall pericardial disease. Overall pericardial disease grouped patients experiencing pericarditis, pericardial effusion, cardiac tamponade, and general non-specific pericardial diseases. For each survival comparison, eligible patients were selected using 1:1 propensity score matching, using a nearest neighbors approach with a caliper width of 0.1. Matching considered the following variables: age, sex, race/ethnicity, neoplasm type, presence of secondary malignancies, hypertension, hyperlipidemia, diabetes mellitus, ischemic heart diseases, heart failure, cerebrovascular diseases, kidney diseases, nervous system diseases, respiratory system diseases, and digestive tract diseases (Supplemental Table 1). One-year survival rates were reported as hazard ratios; p-values from log-ranked statistical tests less than 0.05 were considered significant. In the same way described for incidence determination, survival comparisons of matched populations excluded patients having any diagnosis history of each cardiac irAE considered event prior to ICI initiation.

Each analysis conducted in this study was through the TriNetX analytics platform. This is including incidence determinations, population matching for survival comparisons, and Kaplan Meier survival curves with Cox-proportional hazards statistics. Through our association with the University of California, Irvine, we were granted access to the TriNetX analytics platform, which is not freely accessible without paid subscription. This study, using de-identified data, was exempt from ethical approval.

## Results

**Fig. 1:**
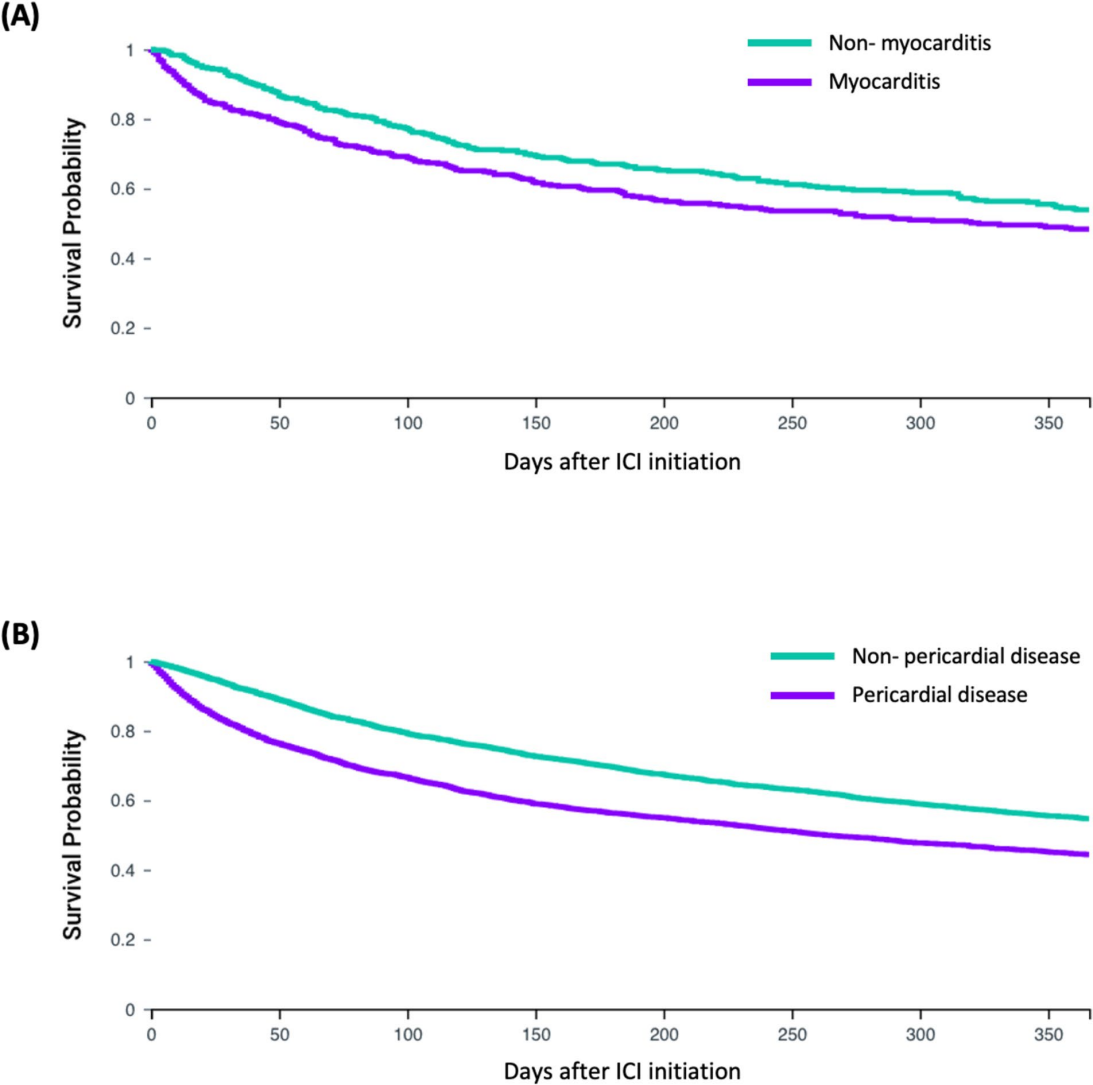
Kaplan Meier Survival Curves Comparing 1 Year Overall Survival Patients Experiencing Myocarditis and Pericardial diseases. **A** represents myocarditis patients (*n* = 424) and matched non-myocarditis patients (HR:1.26, 95% CI:1.04–1.54, *p* = 0.02). **B** represents pericardial disease patients and matched non-pericardial disease patients (*n* = 3,945, HR:1.46, 95% CI:1.37–1.56, *p* < 0.0001). A.O. and M.S. conceived and designed the study; A.O., M.S., and H.H acquired and performed data analysis; A.O, M.S, H.H, M.N., B.L., J.D., A.N., N.N., Y.Z., and P.P. interpreted the results, drafted, and revised the final manuscript

In this study of 88,928 patients treated with ICIs, cardiac inflammatory AEs had incidence proportions below 1% for myocarditis (0.48%), pericarditis (0.22%), and cardiac tamponade (0.47%). Pericardial effusion occurred in 4.71% of the patients (Supplemental Table 2). Hazard ratios (HRs) for OS were significantly higher for each cardiac inflammatory AE compared to matched non-AE cohorts: myocarditis (HR:1.26, 95% CI:1.04–1.54, *p* = 0.02), pericarditis (HR:1.36, 95% CI:1.02–1.82, *p* = 0.04), pericardial effusion (HR:1.49, 95% CI:1.39–1.59, *p* < 0.0001), cardiac tamponade (HR:2.15, 95% CI:1.79–2.57, *p* < 0.0001), and overall pericardial disease (HR:1.46, 95% CI:1.37–1.56, *p* < 0.0001) (Fig. 1, Table 2, Supplemental Figs. 1–3). There was no significant difference in OS between myocarditis and pericarditis or overall pericardial diseases (Table 2, Supplemental Figs. 4–5).

**Table 2:** One-year overall survival models utilized. From Left to write, the first two columns represent the two comparison populations for the survival analysis conducted. Following this, the matched population sizes and number of morality events in each comparison group are represented. Finally, the median survival time for each comparison group are represented followed by the hazard ratio comparing survival probability and its associated p-value. The hazard ratio with its associated 95% confidence interval below it (in parenthesis). Hazard ratios are reported relative to group 1 (ie Values greater than 1 suggest group 1 has increased 1 year mortality risk relative to group 2)

Analysis Conduced		Matched Population Sizes	Mortality Events (n)		Median Survival Time (days)		Hazard Ratio, (95% CI)	*P*-value
Group 1	Group 2		Group 1	Group 2	Group 1	Group 2		
Myocarditis	Non- myocarditis	424	204	185	323	*Majority alive*	1.26, (1.04- 1.54)	0.02
Pericarditis	Non- pericarditis	192	101	85	249	*Majority alive*	1.36, (1.02- 1.81)	0.04
Tamponade	Non- tamponade	415	288	204	84	324	2.15, (1.79- 2.57)	< 0.01
Pericardial effusion	Non- pericardial effusion	3,756	1,928	1,542	266	*Majority alive*	1.49, (1.39- 1.59)	< 0.01
Pericarditis Disease	Non- pericardial disease	3,945	2,027	1,659	263	*Majority alive*	1.46, (1.37- 1.56)	< 0.001
Myocarditis	Pericarditis	144	69	75	267	249	0.97, (0.70- 1.35)	0.87
Myocarditis	Pericardial Disease	376	184	191	289	251	0.95, (0.77- 1.16)	0.60

## Discussion

Our study provides a comprehensive view of ICI-induced myocardial and pericardial inflammatory AEs with a uniquely large cohort. While previous research has reported a wide incidence range for ICI-induced myocarditis (from < 0.1% to 2%), our extensive sample size affirms an incidence of less than 1% [5]. Notably, our findings also expand on the limited data available for pericardial diseases, showing their incidence to be comparably rare except pericardial effusion [6]. These events significantly reduced survival time for patients who developed cardiotoxicity from ICIs, which was expected for myocarditis given available literature. No significant difference was observed in survival time for patients experiencing pericardium-based adverse events compared to myocarditis.

Demonstrating pericardial cardiotoxicity from ICIs having similar mortality implications as myocarditis has important inferences. Diagnosing myocarditis versus pericarditis can be challenging due to varied clinical presentation [7]. Myocarditis and pericarditis can occur concomitantly, and testing with biomarkers (i.e., high-sensitivity cardiac troponin, C-reactive protein) or ECG interpretation can be inconclusive[8]. Given this, early detection strategies can improve patient outcomes. Our study underlines the importance of vigilance for signs of myocardial or pericardial involvement in ICI-treated patients, prompting refinement of monitoring protocols.

Retrospective data base studies do have limitations to consider. Residual confounding may influence results despite extended propensity score matching. Using diagnostic codes to identify cardiac inflammatory AEs may underestimate incidence if events are underdiagnosed or coded incorrectly. Excluding patients with prior cardiac inflammatory events might have eliminated those at highest risk, potentially underestimating the incidence and impact of these events post-ICI therapy. There are various matching criteria that would have made our analysis more robust that simply not available; Future researchers can explore further criteria such as explicit cancer staging information, year of initiation, or other patient demographic factors (IE BMI or familial history). Furthermore, within the platform competing risk analysis was not available which may have provided additional insights. Within a cohort of cancer patients, mortality risk can come from many different factors. Future efforts can implement competing risk survival models. Nonetheless, our diverse cohort provides robust estimates of the incidence and survival impact of rare cardiac inflammatory AEs.

## Conclusion

With the largest cohort studied to date, our findings provide valuable insights into the incidence and survival impact of rare but clinically significant cardiac inflammatory AEs associated with ICI therapy. While myocarditis and pericarditis presented with similar incidence rates and associated mortality, pericardial effusion was more prevalent and equally severe. These insights emphasize the necessity for clinicians to closely monitor for early signs of cardiac involvement. This study reaffirms the importance of vigilance regarding cardiac irAEs and lays groundwork for future research on predictive models and preventative strategies for such adverse events in ICI-treated patients.

## Supplementary Information

Supplementary Material 1.

## Data Availability

The data that support the findings of this study are available from TriNetX but restrictions apply to the availability of these data, which were used under license for the current study, and so are not publicly available. Data are however available from the authors upon reasonable request and with permission of TriNetX.

## Abbreviations

AEs: Adverse events
CI: Confidence interval
HR: Hazard ratio
ICIs: Immune checkpoint inhibitors
irAE: Immune-related adverse events
OS: Overall survival
