# Supplementary material for: Incidence and survival outcomes of myocarditis and pericardial diseases associated with immune checkpoint inhibitor therapy
Source: Cardiooncology. 2025 Mar 5;11:26. doi: 10.1186/s40959-025-00300-1 (PMC11881249; doi:10.1186/s40959-025-00300-1)
Supplement: Supplementary file 1 — Supplementary Material 1. [file 40959_2025_300_MOESM1_ESM.docx]

**Supplemental File:**

**Supplemental Table 1: Criteria Utilized For Cohort Matching**

Matching criteria are organized in to groups of demographic data, cancer diagnosis history, and comorbid conditions indicated by bolded headers. Indented entries below each header represent matching criteria utilized within the Trinetx analytics platform, with the criteria names identical the platforms names. For relevant criteria, the ICD-Codes they represent are listed below them.

| **Demographic Data** |
| --- |
| Age at ICI Index |
| Sex |
| Race/Ethnicity: White, Unknown Race, Unknown Ethnicity, Not Hispanic or Latino, Hispanic or Latino, Black or African, Asian |
| **Cancer Diagnosis History** |
| Malignant neoplasms of respiratory and intrathoracic organs  ICD-Codes: C30- C39 |
| Melanoma and other malignant neoplasms of skin  ICD-Codes: C43- C44 |
| Malignant neoplasms of urinary tract  ICD-Codes: C64- C68 |
| Malignant neoplasms of breast  ICD-Codes: C50 |
| Secondary malignant neoplasm of respiratory and digestive organs  ICD-Codes: C78 |
| Secondary and unspecified malignant neoplasm of lymph nodes  ICD-Codes: C77 |
| Malignant neoplasm without specification of site  ICD-Codes: C80 |
| Malignant neoplasms of lymphoid, hematopoietic, and related tissue  ICD-Codes: C81- C96 |
| Malignant neoplasms of digestive organs  ICD- Codes: C15- C26 |
| Malignant neoplasm of prostate  ICD- Codes: C61 |
| **Comorbid Conditions** |
| Heart Failure  ICD-Codes: I50 |
| Ischemic heart diseases  ICD-Codes: I20- I25 |
| Cerebrovascular diseases  ICD-Codes: I60- I69 |
| Diabetes mellitus  ICD-Codes: E08- E13 |
| Diseases of the digestive system  ICD-Codes: K00- K95 |
| Diseases of the respiratory system  ICD-Codes: J00- J99 |
| Diseases of the nervous system  ICD-Codes: G00- G99 |
| Chronic Kidney Disease  ICD-Codes: N18 |
| Hyperlipidemia, unspecified  ICD-Codes: E78.5 |

**Supplemental Table 1: Incidence of different cardiac inflammatory immune related adverse events**

From left to right, the first column represents the type of cardiac inflammatory immune related adverse event, followed by the total number of patients experiencing the event having no diagnosis history of the diagnosis prior to ICI initiation. The total patients column represents the total number of ICI patients having no history of the irAE of interest prior to ICI initiation, followed last by the calculated incidence represented as a percentage.

|  | Total Events | Total  Patients | Incidence (%) |
| --- | --- | --- | --- |
| Myocarditis | 424 | 88928 | 0.48 |
| Pericarditis | 194 | 88806 | 0.22 |
| Cardiac Tamponade | 415 | 88549 | 0.47 |
| Pericardial Effusion | 3937 | 83617 | 4.71 |
| Pericardial Disease | 4135 | 82947 | 4.99 |


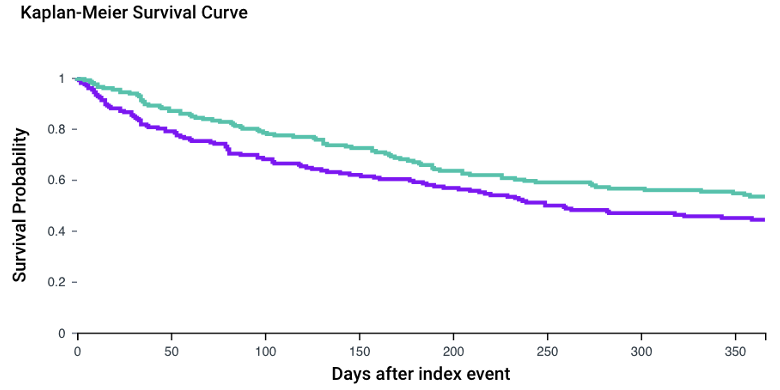


**Supplemental Figure 1: Kaplan Meier survival curve comparing survival of immune checkpoint inhibitor patients experiencing pericarditis against those that do not.**

Patients experiencing pericarditis are represented as the purple line while those that did not are represented by the teal blue line (HR: 1.36, CI: 1.02- 1.81, p= 0.04).


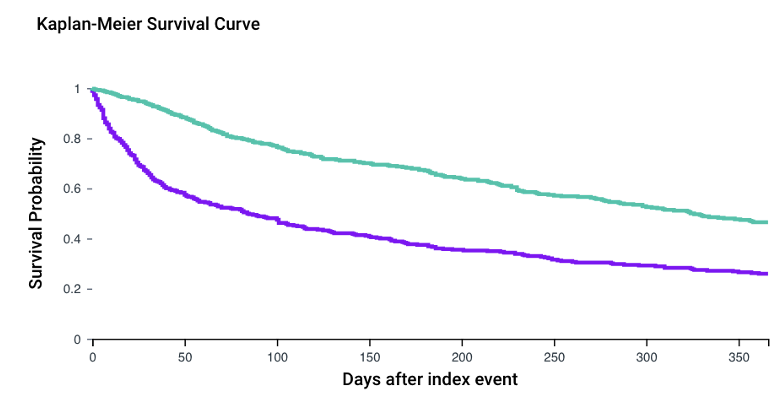


**Supplemental Figure 2: Kaplan Meier survival curve comparing survival of immune checkpoint inhibitor patients experiencing cardiac tamponade against those that do not.**

Patients experiencing cardiac tamponade are represented as the purple line while those that did not are represented by the teal blue line (HR: 2.15, CI: 1.79- 2.57, p= <0.01).


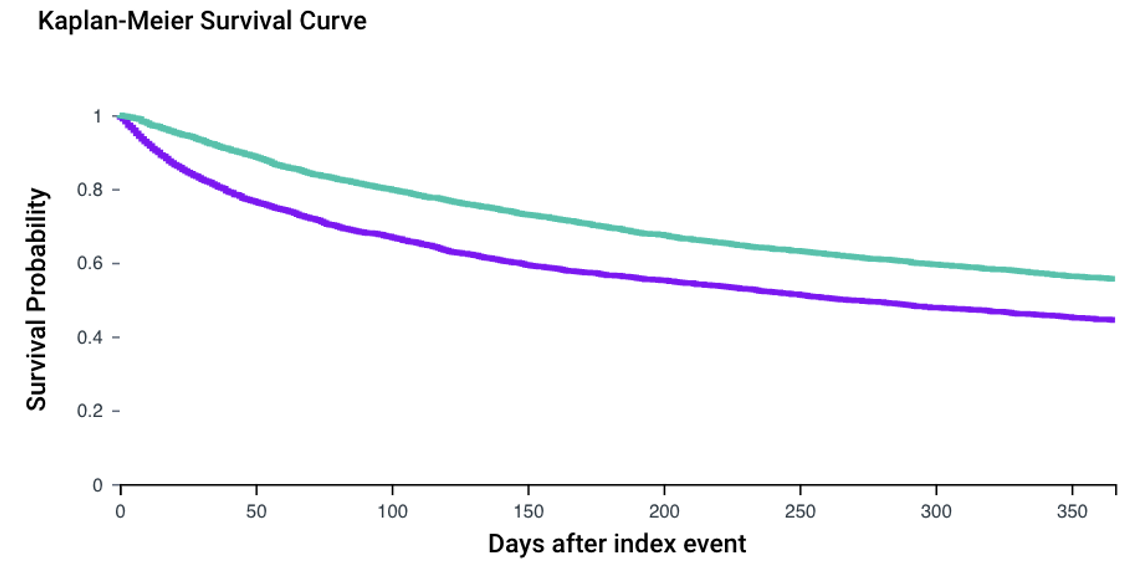


Supplemental Figure 3: **Kaplan Meier survival curve comparing survival of immune checkpoint inhibitor patients experiencing pericardial effustion against those that do not.**

Patients experiencing cardiac pericardial effusion are represented as the purple line while those that did not are represented by the teal blue line (HR:1.49, CI:1.39-1.59, p= <0.01).


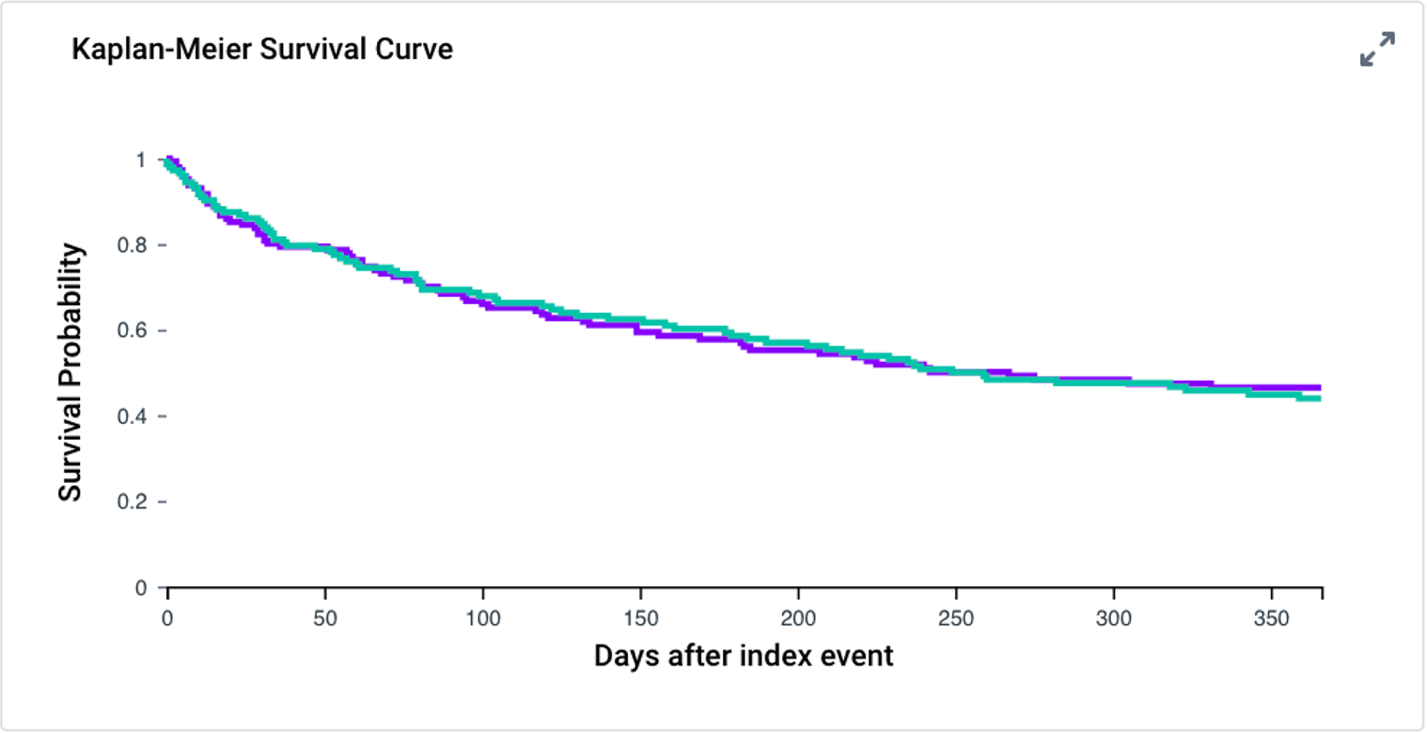


Supplemental Figure 4: **Kaplan Meier survival curve comparing survival of immune checkpoint inhibitor patients experiencing myocarditis against those that experience pericarditis.**

Patients experiencing myocarditis are represented as the purple line while those that experienced pericarditis are represented by the teal blue line (HR: 0.97, CI: 0.70- 1.35, p= 0.87).


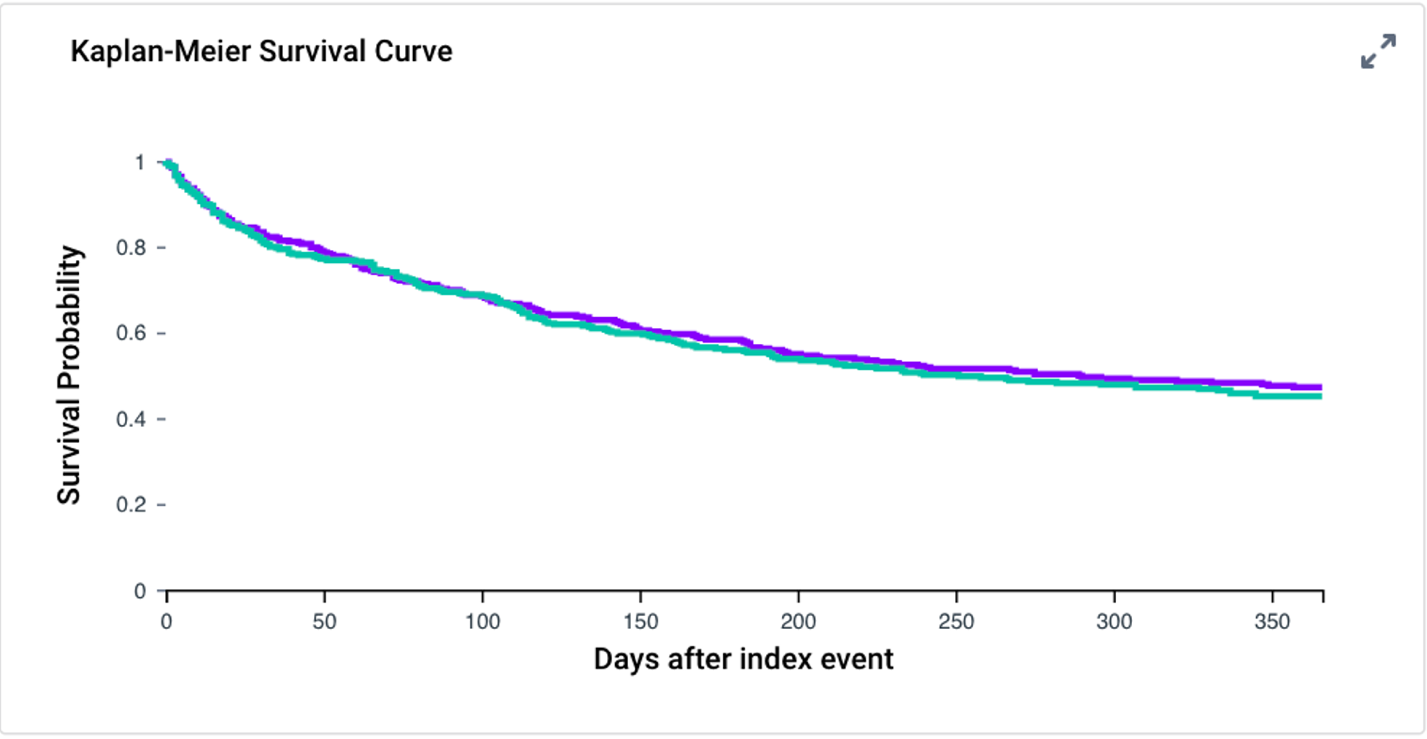


**Supplemental Figure 5: Kaplan Meier survival curve comparing survival of immune checkpoint inhibitor patients experiencing myocarditis against those that experience overall pericardial disease.**

Patients experiencing myocarditis are represented as the purple line while those that experienced pericardial disease are represented by the teal blue line (HR:0.95, CI: 0.77- 1.16, p= 0.60)
